# Inflammation significantly alters mucosal transcriptomic signatures in pediatric inflammatory bowel disease

**DOI:** 10.1093/crocol/otag023

**Published:** 2026-03-28

**Authors:** Alexander Schnell, Xinyi Wei, Jan Bossenz, Iryna Manuilova, Jan Christoph, Ida Allabauer, Merle Classen, Adrian P Regensburger, Joachim Woelfle, Ramona Erber, André Hoerning

**Affiliations:** Pediatric Gastroenterology and Hepatology, Department of Pediatrics and Adolescent Medicine, University Hospital Erlangen, Friedrich-Alexander-University Erlangen-Nuremberg, Erlangen, Germany; Pediatric Gastroenterology and Hepatology, Department of Pediatrics and Adolescent Medicine, University Hospital Erlangen, Friedrich-Alexander-University Erlangen-Nuremberg, Erlangen, Germany; Department of Obstetrics and Gynecology, The Third Affiliated Hospital of Zhengzhou University, Zhengzhou, China; Junior Research Group (Bio-)medical Data Science, Faculty of Medicine, Martin-Luther-University Halle-Wittenberg, Halle, Germany; Junior Research Group (Bio-)medical Data Science, Faculty of Medicine, Martin-Luther-University Halle-Wittenberg, Halle, Germany; Junior Research Group (Bio-)medical Data Science, Faculty of Medicine, Martin-Luther-University Halle-Wittenberg, Halle, Germany; Pediatric Gastroenterology and Hepatology, Department of Pediatrics and Adolescent Medicine, University Hospital Erlangen, Friedrich-Alexander-University Erlangen-Nuremberg, Erlangen, Germany; Department of Pediatrics and Adolescent Medicine, University Hospital Erlangen, Friedrich-Alexander-University Erlangen-Nuremberg, Erlangen, Germany; Pediatric Gastroenterology and Hepatology, Department of Pediatrics and Adolescent Medicine, University Hospital Erlangen, Friedrich-Alexander-University Erlangen-Nuremberg, Erlangen, Germany; Department of Pediatrics and Adolescent Medicine, University Hospital Erlangen, Friedrich-Alexander-University Erlangen-Nuremberg, Erlangen, Germany; Institute of Pathology, University Hospital Erlangen, Friedrich-Alexander-University Erlangen-Nuremberg, Erlangen, Germany; Pediatric Gastroenterology and Hepatology, Department of Pediatrics and Adolescent Medicine, University Hospital Erlangen, Friedrich-Alexander-University Erlangen-Nuremberg, Erlangen, Germany

**Keywords:** pediatric IBD, DCA score, RNA sequencing, pediatric IBD, DCA score, RNA sequencing

## Abstract

**Objectives and study:**

Crohn’s disease (CD) and ulcerative colitis (UC) share common features of inflammation to a greater extent in children than in adults. However, histopathological scoring systems of mucosal inflammation are usually available only for either one of these entities. The IBD-DCA score is the first of its kind to incorporate both into one scoring system but still lacks validation in pediatric IBD.

**Methods:**

An existing data of a multiplexed gene expression analysis of mucosal biopsies of 25 patients diagnosed with either CD (*n* = 16) or UC (*n* = 9) were evaluated according to the IBD-DCA score for distribution (D0-2), chronicity (C0-2) and activity (A0-2) of inflammation by an experienced pathologist. The scoring results were used to stratify the degree of mucosal inflammation of these patients into either low (0-3) or high (4-6) DCA scores. Subsequently, analysis for differentially expressed genes (DEG) between the low and high inflammation group was performed.

**Results:**

Scoring revealed 7 low and 9 high DCA samples for CD, whereas for UC only high DCA were scored. DEG analysis revealed 130 upregulated genes in the high DCA group compared to the low DCA group. 11 genes were identified as hub genes playing a pivotal role in immune regulation, among those several cyto- or chemokines (IL1B, CCL20, CXCL1/2), costimulatory receptors (IL2RA, CD80, TLR2) and modulatory proteins like PD-L1 and IDO1.

**Conclusion:**

Our results indicate that inflammatory activity rather as assessed by the IBD-DCA score rather than the underlying disease entity significantly alters the transcriptomic signature in mucosal specimens of pediatric IBD patients.

## Graphical Abstract

**Graphical Abstract:**
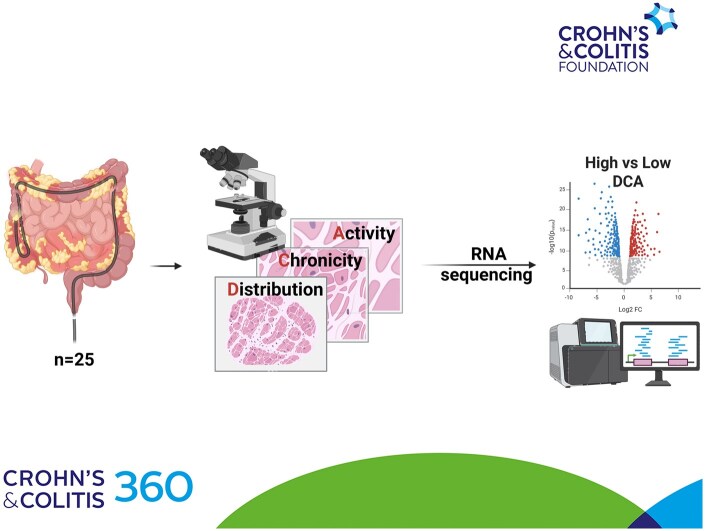

## Introduction

Inflammatory bowel disease (IBD) represents a challenging spectrum of chronic gastrointestinal disorders that significantly impact the health and quality of life of affected individuals, particularly in pediatric populations. Crohn’s disease (CD) and ulcerative colitis (UC), the two main subtypes of IBD, are characterized by chronic inflammation of the gastrointestinal tract.^1^ In contrast to adults, where both diseases represent two distinct entities, pediatric IBD often display overlapping features of CD and UC, for example, isolated colonic disease in children with CD,^2–4^ with the consequence that a diagnosis can sometimes only be found in the process of histopathological work-up. Apart from that, histopathological evaluation also plays a crucial role in the management of pediatric IBD, providing valuable insights into disease activity, severity, and mucosal healing.^5^ This has particularly become important, as advancements in therapeutic strategies have pushed the limits for treatment goals from clinical to mucosal or even molecular remission.^6^ To accomplish that, standardized and validated scoring tools are needed that are able to assess inflammatory activity and are ideally applicable in CD and UC alike. To date, there are >30 histopathological scores for UC, however only the Nancy Index and Robarts Histopathological Score have been—at least—partially validated, but complete disregard “architectural” features like crypt configuration^7^ which is an important discriminator between quiescent UC and complete histological normalization.^8^ For CD, none of the existing 13 scores have been validated.^9^

Among those various histopathological scoring systems, the IBD-DCA histopathological score has been emerged in 2021 as a promising tool specifically tailored for meeting those needs.^10^ The IBD-DCA score has been developed to incorporate an easily feasible scoring tool in the regular work flow of a histopathologist,^11^ thus offering a standardized and validated framework for assessing the histopathological features and grading disease severity in pediatric patients of both entities.

This study aims to explore the significance and usability of the IBD-DCA histopathological score in pediatric IBD for categorizing our patient cohort according to the degree of mucosal inflammation. Here, we further investigated mucosal RNA molecular patterns that are associated with inflammatory activity on a subcellular level, thus contributing to a deeper understanding of IBD in this vulnerable patient population.

## Materials and methods

### Patients and samples

For this study, children suspected or diagnosed with pediatric IBD undergoing diagnostic colonoscopy were recruited. The study was approved by a scientific ethics committee (#347_15B), all participants provided informed consent prior to study enrollment.

At least two to three intestinal biopsies were taken from the terminal ileum, ascending colon, transversal colon, descending colon, sigma, and rectum, and one biopsy from the caecum as part of the routine diagnostics and sent to the Institute of Pathology for histopathological workup.

Patient demographic data are shown in Table 1. After endoscopy and histopathological analysis, all patients fulfilled the ECCO criteria for Crohn’s disease (CD) or ulcerative colitis (UC). Therapy decisions were not influenced by results of this study in any way.

**Table 1:** Characteristics of the study cohort.

Disease	Age	Paris classification	Biopsy localization	Distribution	Chronicity	Activity	DCA score	DCA cohort	Calpro µg/g	UCEIS/SES-CD	PUCAI/PCDAI	PUCAI/PCDAI	Treatment
***UC1***	13	E2	Sigma	2	2	1	5	High	341	4	30	Mild	TAC, 5-ASA
***UC2***	15	E3	Colon transv.	2	2	2	6	High	87 998	6	35	Moderate	Aza, 5-ASA
***UC3***	15	E2	Colon desc.	2	2	2	6	High	85	5	35	Moderate	Aza, P, 5-ASA
***UC4***	16	E4	Colon transv.	2	2	2	6	High	5896	6	55	Moderate	
***UC5***	11	E4	Colon asc.	2	2	2	6	High	81 354	6	55	Moderate	Aza, 5-ASA
***UC6***	13	E4	Colon transv.	2	2	2	6	High	3019	5	70	Severe	
***UC7***	9	E4	Rectum	2	2	2	6	High	464	4	30	Mild	
***UC8***	12	E4	Colon transv.	2	2	2	6	High	176	5	40	Moderate	
***UC9***	16	E4	Sigma	2	2	2	6	High	7458	5	80	Severe	P, 5-ASA
***CD1***	6	L2/L4 B1	Sigma	2	2	2	6	High	190	6	22.5	Mild	P, 5-ASA
***CD2***	13	L3/L4 B1	term. leum	2	2	2	6	High	1642	1	17.5	Mild	Aza, 5-ASA
***CD3***	14	L3/L4 B3p	term. leum	2	2	2	6	High	794	4	47.5	Moderate	Aza, P, 5-ASA
***CD4***	16	L3/L4 B1p	term. leum	2	2	2	6	High	655	6	52.5	Severe	P, 5-ASA
***CD5***	13	L3/L4 B1	term. Ileum	2	2	2	6	High	4932	2	25	Mild	
***CD6***	15	L3/L4 B1p	term. Ileum	2	2	2	6	High	5829	6	57.5	Severe	P
***CD7***	11	L2/L4 B2/3	Colon transv.	2	2	2	6	High	18 315	9	67.5	Severe	
***CD8***	16	L3 B2p	Colon asc.	2	2	0	4	High	4665	9	42.5	Moderate	
***CD9***	1	L2 B1	Coecum	2	2	1	5	High	476	3	27.5	Mild	
***CD10***	15	L2 B1	Colon transv.	2	1	0	3	Low	9.2	n/a	22.5	Mild	P
***CD11***	13	L3/L4 B3p	Colon transv.	2	1	0	3	Low	4018	6	25	Mild	
***CD12***	11	L3/L4 B2	Coecum	2	1	0	3	Low	577	1	32.5	Moderate	
***CD13***	16	L2/L4 B2	Colon transv.^a^	0	0	0	0	Low	41 954	4	50	Severe	
***CD14***	15	L3 B3	Rectum	2	1	0	3	Low	5879	3	17.5	Mild	
***CD15***	16	L3/L4 B2	Sigma	0	0	0	0	Low	369	0	42.5	Moderate/severe	
***CD16***	17	L1 B2/3	Colon asc.^b^	2	1	0	3	Low	91	0	52.5	Severe	

Abbreviations: 5-ASA: 5-aminosalicylate; Aza, azathrioprin; P, prednisolon; TAC, tacrolimus.

a Severe stenosis in ascending colon, therefore sampling was performed distally of the stenosis.

b Patient underwent ileocecal resection prior to colonoscopy.

### RNA extraction

FFPE from the segments with prominent inflammation (as outlined in the routine endoscopic and histopathological reports) was selected for RNA sequencing. mRNA was extracted from FFPE blocks of the same intestinal specimens as for DCA scoring using the RNeasy FFPE Kit (Qiagen, Hilden, Germany). The purity and quality of the extracted RNA were assessed by a Thermo Fisher Scientific spectrophotometer (Waltham, MA, USA). The A260/A280 ratio fell within the range of 1.7–2.3, and the A260/A230 ratio was between 1.8 and 2.3.

### Histopathological scoring using the IBD-DCA score

For stratification of the inflammatory activity within the analyzed samples, the degree of inflammation within each respective fraction of the same patient was retrospectively analyzed by an experienced pathologist with >10 years of practice using the IBD-DCA score as described previously.^10^^,^^11^ On the basis of the scoring results, the specimens were then assigned to one of the following groups for further bioinformatical analysis: DCA-Low for low degrees of inflammation (<4 points) or DCA-High (inflammation) for scoring results between 4 and 6 points.

IBD-DCA scoring was performed for all available fractions from the same patient and endoscopic procedure. All scoring results are summarized in Table S1.

### Activity indices and endoscopic activity scores

For estimating the individual disease activity for each patient, the indices Pediatric Ulcerative Colitis Activity Index (PUCAI) for UC or Pediatric Crohn’s Disease Activity Index (PCDAI) was collected as described.^12–14^ Endoscopic activity was assessed retrospectively from the stored image data of each colonoscopy using the Ulcerative Colitis Endoscopic Index of Severity (UCEIS) for UC^15^ and the Simple Endoscopic Score for Crohn’s Disease (SES-CD) for CD.^16^

### NanoString biostatistical analysis and identification of differentially expressed genes (DEGs)

An expression profile of 594 immune-related genes was performed using the nCounter^®^ Human Immunology v2 Panel (XT-CSO-HIM2-12). The raw data was processed and quality controlled using nSolver Analysis Software version 4.0 (NanoString Technologies Inc., Seattle, Washington, USA). Normalization of the raw data was conducted using positive control genes (POS_A, POS_B, POS_C, POS_D, POS_E, and POS_F); the housekeeping genes (ABCF1, ALAS1, EEF1G, G6PD, GAPDH, GUSB, HPRT1, OAZ1, POLR1B, POLR2A, PPIA, SDHA, TBP, TUBB, and RPL19); and the geNorm module of the advanced nSolver analysis 4.0 software (MAN-C0019-08). *P* values were corrected for multiple hypotheses testing using the False Discovery Rate method by Benjamini-Yekutieli.^17^ The adjusted *P*-values (adj. *P*) <.05 and Log2 fold-change (FC) >1 were considered differentially expressed genes. Heatmaps were generated using the Heatmap V1.0.12 package^18^ in the R software statistical analysis platform. Principal component analysis (PCA) was conducted to visualize sample clusters by the Scatterplot3d V0.3-42 package in R software. A volcano plot was created by GraphPad Prism 9.4.0 to display −log10 *P*-value and Log2FC for each gene.

### Construction of protein–protein interaction (PPI) network and identification of hub genes

We used STRING V11.5 (http://www.string-db.org/),^19^^,^^20^ an online tool designed for identifying and predicting interactions between genes or proteins, to construct a PPI network of DEGs. The cut-off standard was considered an interaction score greater than 0.4 (medium confidence). Subsequently, we employed Cytoscape V3.9.1.^21^ to visualize the protein-protein interaction network of DEGs. Following that, we utilized Cytohubba V0.1,^22^^,^^23^ another Cytoscape plugin, to analyze essential nodes in the PPI network and select hub genes using various algorithms.

### Use of the large language model OntoGPT

The large language model (LLM) OntoGPT was utilized to assign at least one of the parameters (D, C, and A) to each gene from the DEG list. For each gene, a separate query was generated, which included a detailed description of the gene, associated biological process (GO), molecular function (GO), and an explanation of the respective parameters of the DCA score based on the publication by Schwarz et. al.^11^ The OntoGPT command “complete” was employed, using the “gpt-4” model for processing. Additionally, the desired output format was specified within the query.

To enhance the decision-making process of the LLM, the description of the DCA parameters was further refined and specified during the process. Despite these refinements, OntoGPT encountered difficulties in assigning a quantitative value to each gene. Consequently, the query format was adjusted and a qualitative rating on a five-point Likert scale (strongly applicable, applicable, neutral, not applicable, not applicable at all) was requested for each of the three parameters.

The queries for all genes in the DEG list were executed automatically using a bash script. These results were then compared with a manually curated assignment list to evaluate the quality of the automated assessments.

## Results

### Clinical and demographic characteristics of IBD patients

Twenty-five patients with diagnosed IBD (UC: *n* = 9; CD: *n* = 16) were included for this study. Analysis of the individual activity indices revealed a predominance of moderate-to-severe disease activity (PUCAI: 47.7 ± 17.2; PCDAI: 37.6 ± 15.3) with elevated concentrations of fecal calprotectin for both entities (UC: 22 416 µg/g, range 85-87 998 µg/g; CD: 6416 µg/g, range 9-18 315 µg/g). The characteristics of each patient are summarized in Table 1.

**Figure 1:**
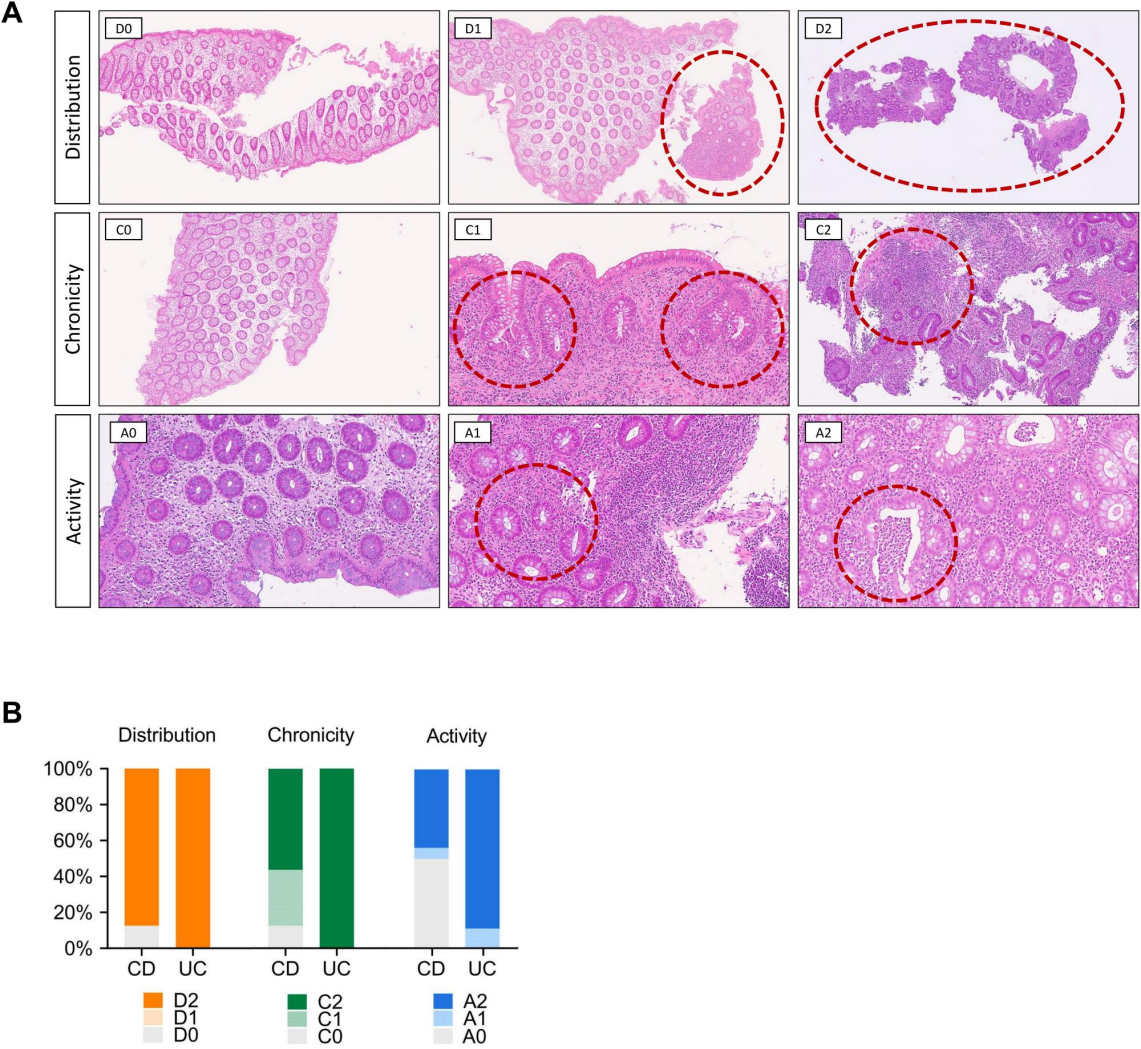
IBD-DCA score and Histological features of IBD patients. (A) Representative images for each parameter and severity. DCA scoring according to [Lang-Schwarz, VIAR 2020] in formalin fixed, paraffin embedded and hematoxylin & eosin stained biopsies of the large intestine: Distribution: D0) No mucosal abnormalities (100×); D1) mucosal abnormalities in <50% of the tissue (dotted line; 100×); D2) mucosal abnormalities in ≥50% of the tissue (dotted line; 50×). Chronicity: C0) No chronic changes (100×); C1) crypt distorsion (dotted line; 400×) and mild lymphoplasmocytosis in lamina propria; C2) marked lymphoplasmocytosis in lamina propria (dotted line; 200×). Activity: A0) No neutrophilic granulocytes within the mucosa (400×); A1) ≥2 neutrophils in the lamina propria per one high power field (HPF) or ≥1 neutrophil(s) in the epithelium (here both applies (dotted line); 400×); A2) crypt abscess′s (dotted line; 400×), erosion or ulceration. (B) Percentage of different score of distribution, chronicity, and activity by IBD cohorts (CD and UC patients). Abbreviations: IBD, inflammatory bowel disease; IBD-DCA score, inflammatory bowel disease—distribution, chronicity, activity score; CD, Crohn′s disease; UC, ulcerative colitis.

**Figure 2:**
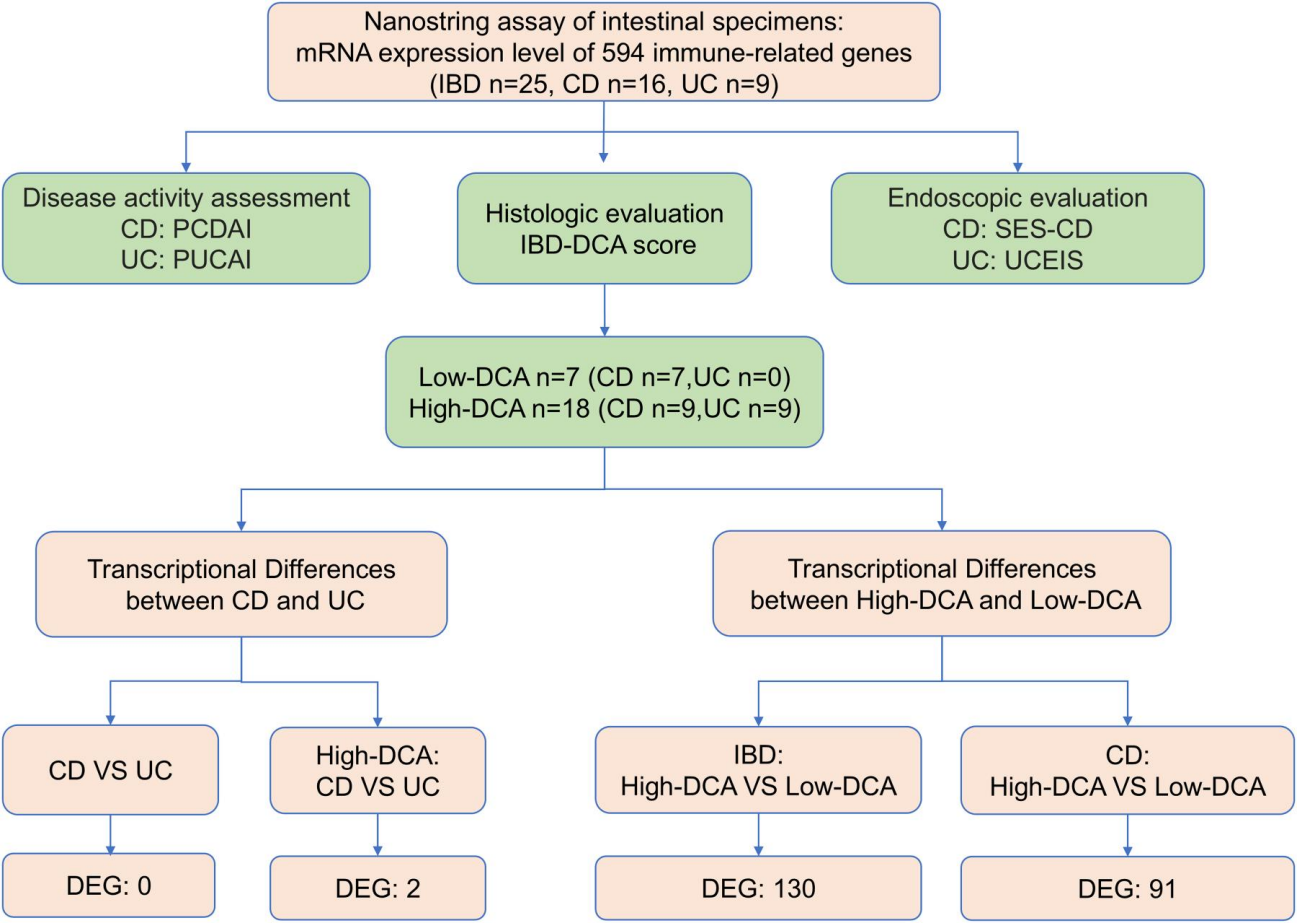
Study flow. Abbreviations: IBD, inflammatory bowel disease; IBD-DCA Score, inflammatory bowel disease—distribution, chronicity, activity score; CD, Crohn′s disease; UC, ulcerative colitis.

Histopathological evaluation showed a consistently increased degree of mucosal inflammation for all patients with UC, whereas patients with CD displayed a more heterogeneous picture (Table 1). Figure 1 shows representative HE stainings highlighting the DCA analysis of the IBD cohort by demonstrating each specific DCA parameter. Accordingly, all patients with UC were assigned to the DCA-High group. In the CD cohort, IBD-DCA scoring of the previously selected specimens of seven patients displayed rather mild or absence of inflammation and were therefore assigned to the DCA-Low group. The remaining nine patients showed prominent features of inflammation and were therefore included in the DCA-High group. The complete study flow is summarized in Figure 2.

### Comparison of intestinal immune transcriptomes in IBD patients between the high-DCA group and low-DCA group

**Figure 3:**
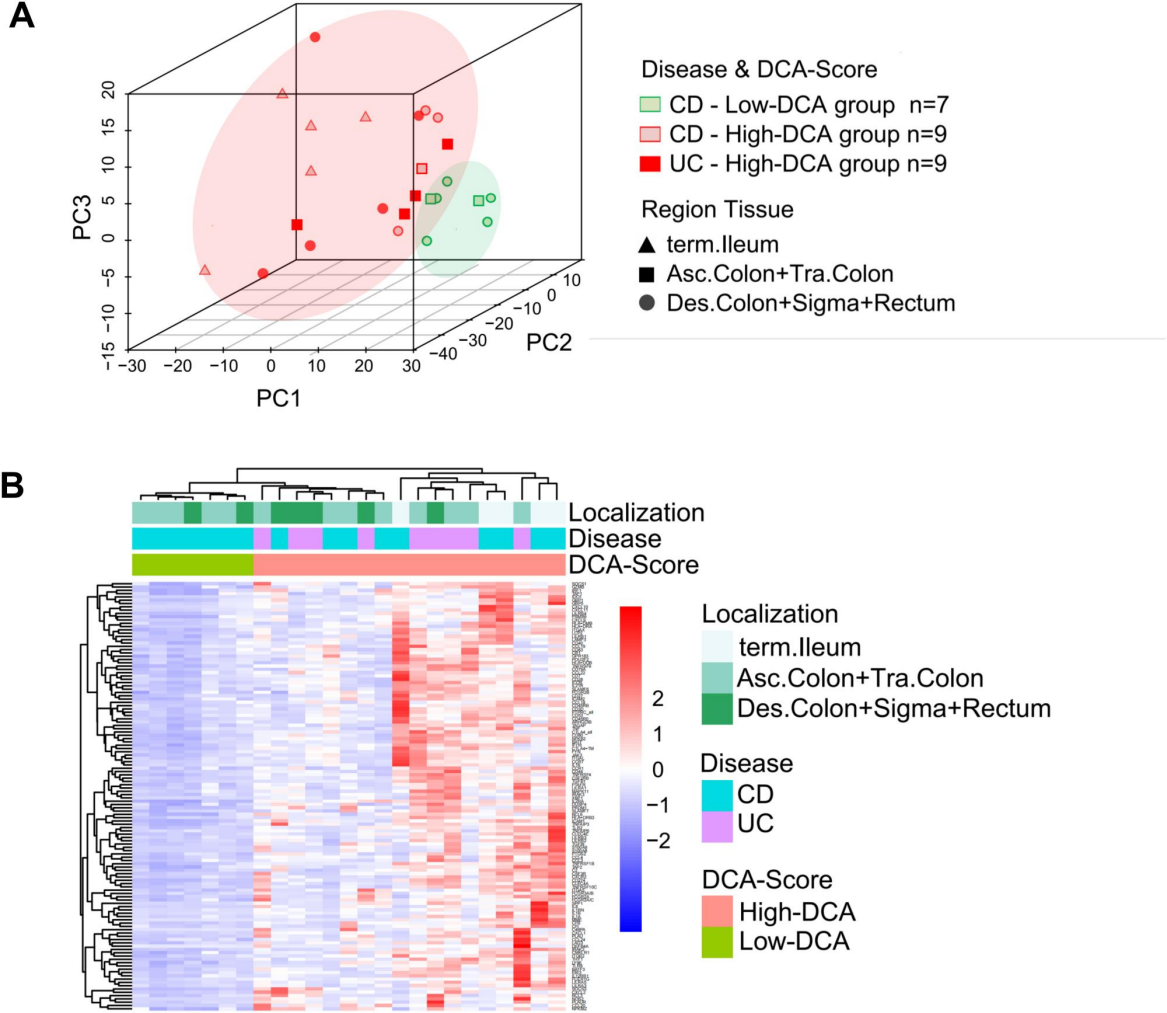
(A) 3D scatterplots of principal component analysis. High DCA: red symbols, low DCA (only CD): green. Region tissue: triangle (terminal Ileum), squares (ascending and transversal colon), circles (descending colon + Sigma + Rectum) (B) Heatmaps were generated based on the 130 differentially expressed genes (adj. *P *< .05) manually clustered for disease entity, biopsy localization and DCA-score. Abbreviations: asc., ascending; CD, Crohn’s disease; desc., descending; term., terminal; tra., transversal; UC, ulcerative colitis.

PCA conducted on all 579 genes (excluding the housekeeping genes) revealed two distinguishable groups, indicating a clear separation between high-DCA and the low-DCA clusters. The cumulative variance explained by the first three principal components (PC1, PC2, and PC3) amounted to 39.92%, 16.16%, and 10.65% of the total variance, respectively (Figure 3A). However, we also observed that the CD and UC groups were closely intertwined and indistinguishable, suggesting a considerable similarity in the immune-related transcriptome expression profiles between patients with Crohn’s disease and ulcerative colitis (Figure 3A).

Furthermore, we conducted further analysis by selecting genes with significant differential expression in the intestinal mucosal transcriptome of IBD patients between the high-DCA and low-DCA cohorts. The resulting heatmap exhibited a clear polarization between the two groups, underscoring the pronounced differences in gene expression patterns associated with DCA levels (adj. *P* < .05; Figure 3B). Notably, also the localization of the respective biopsies as well as the disease entity showed some sort of clustering to a certain degree in the PCA and heatmap plots; however, to a lesser extent than the DCA score results.

### Identification of DEGs between the high-DCA and the low-DCA of IBD patients

**Figure 4:**
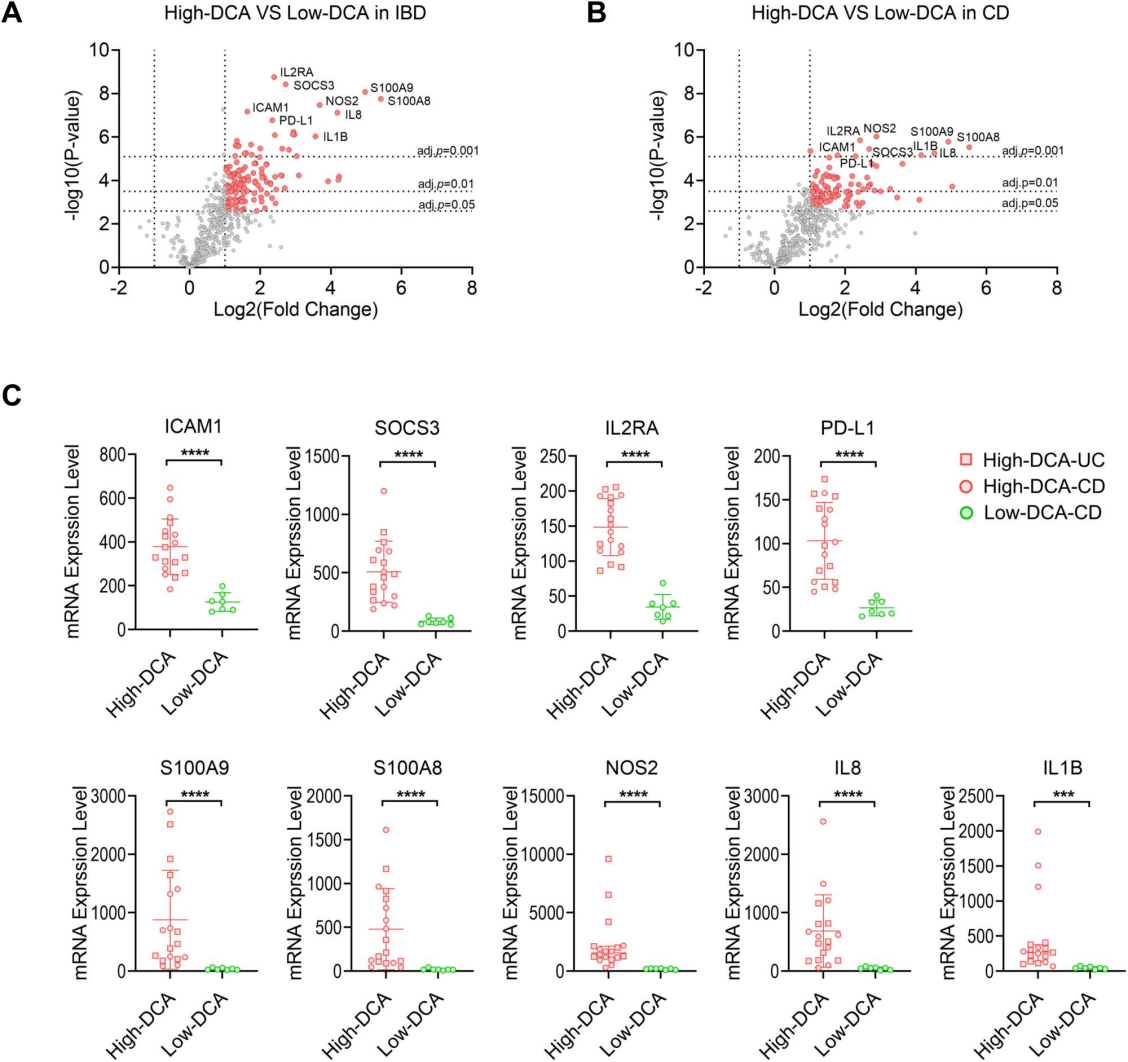
Mucosal RNA expression patterns. (A and B) Volcano plots depict differentially expressed genes when the High-DCA and the Low-DCA group for the entire IBD cohort (A) or only CD patients (B) are compared. Red dots represent significantly elevated expression. The criteria are set as Log FC > 1, adj. *P* < .05. (C) mRNA expression of representative differentially expressed genes between the High-DCA and Low-DCA group of the IBD cohort. Abbreviations: IBD-DCA Score, Inflammatory Bowel Disease—Distribution, Chronicity, Activity Score; CD, Crohn′s disease; UC, ulcerative colitis. ***Adjusted *P* < .001; ****Adjusted *P* < .0001.

To further evaluate, we conducted a deeper analysis of Differentially Expressed Genes (DEGs) and identified a cluster comprising 130 genes exhibiting significant differences in expression levels between the high-DCA group and the low-DCA group (Log2FC > 1, adj. *P* < .05, Table S2). The volcano plot in Figure 4A displayed the distribution of DEGs, revealing 130 upregulated genes and 0 downregulated genes. We observed that among these differentially expressed genes, the most significantly differentially expressed genes include *ICAM1, PD-L1, IL2RA, SOCS3, S100A9, S100A8, NOS2, IL8,* and *IL1B* (adj. *P* < .001). We also performed a separate analysis for the CD cohort only. Here, the DEG analysis provided 91 upregulated genes (Table S2 and Figure 4B) among which 81 DEGs were also present in the analysis for the entire IBD cohort. Only the genes *NFKBIA, RARRES3, CD74, IRF8, ITLN2, NLRP3, HLA-DMB, CASP1, CIITA, STAT1* were differentially expressed specifically in CD.

Figure 4C depicts the normalized mRNA expression levels of the above mentioned, most significant genes for both analyses as assessed by standard Nanostring expression analysis.

Notably, we also performed a differential gene expression analysis between UC and CD patients within the high DCA cohort, but this yielded only two DEGs (RARRES3, DPP4). Moreover, as PCA plot and heatmap analysis revealed a potential influence of the localization where the biopsy was taken, a separate subanalysis in that regard was performed. This revealed a total of 24 DEGs for the terminal ileum compared to the proximal colon (caecum, ascending colon, and transversal colon), and 27 DEGs compared to the distal colon (descending colon, sigma, and rectum) for the entire IBD cohort (Figure S1A). Interestingly, there were only six (proximal) or five (distal) overlapping genes with the analysis high vs low DCA score (Figure S1B). Notably, when we performed the same analysis for CD patients exclusively, the amount of DEGs increased to *n* = 111 for the comparison of the terminal Ileum vs proximal colon, whereas for the distal colon, the results did not change (Figure S1C). In that analysis, 57 out of those 111 DEGs were also overlapping with the DCA analysis.

### Protein–protein interaction network analysis and the DCA annotation

**Figure 5:**
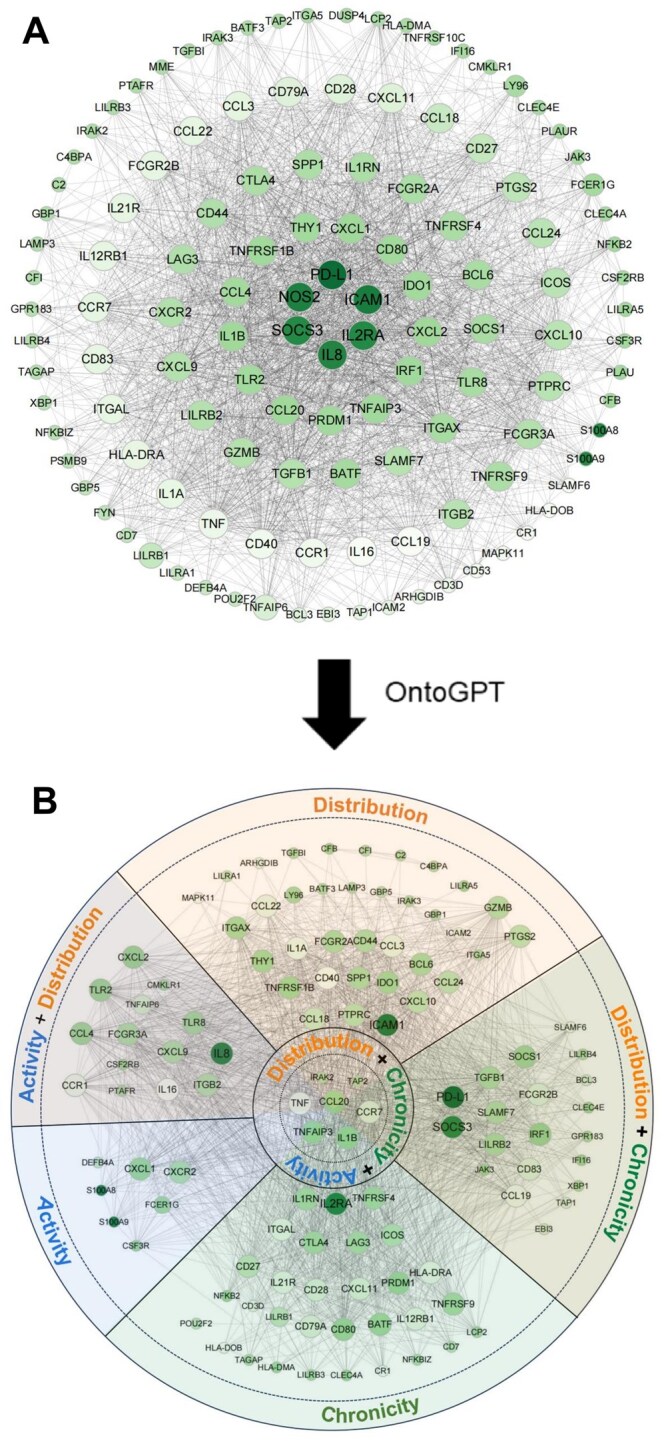
(A) The protein–protein interaction network was processed employing differentially expressed genes identified in between DCA-low and DCA-high patients, The size of the node reflects the degree and intensity of connectivity (MCC), and the larger diameter of the node represents a higher degree. The color intensity represents the adj. *P-*values for the differential expression between the DCA-high and DCA-low groups, with darker colors indicating smaller adj. *P*-values. (B) The differentially expressed genes are displayed in partitions based on the results of DCA annotations. Abbreviations: DCA, distribution, chronicity, activity.

The protein-protein interaction network of DEGs, constructed using STRING, comprised 130 DEGs organized into a cluster with 120 nodes and 1972 edges. The local clustering coefficient was calculated as 0.685, and the protein-protein interaction enrichment *P*-value was <1.0e-16. The network data file was processed and visualized using Cytoscape (Figure 5A). Based on node degree calculations from CytoHubba, we employed the Maximum Clique Centrality (MCC) method to identify hub differentially expressed genes with the highest MCC values. These genes exhibit the highest interaction degree within the protein-protein interaction network (Figure 5A). From these high MCC value genes, we further identified six key genes—ICAM-1, NOS2, SOCS3, IL2RA, IL8, and PD-L1—based on their highly significant differential expression between the DCA-high and DCA-low groups (adj. *P* < .0001, Figure 5A). These genes had the highest connectivity degree within the protein-protein interaction network of differentially expressed genes, indicating they may play a pivotal role in the pathological processes associated with the progression of DCA levels in IBD patients.

Furthermore, we asked the LLM OntoGPT to assign each gene to at least one parameter of the DCA score. Here, the assignment for LLM and the manually curated list (10 genes could not be classified manually) matched for 53/120 genes (44%, Table S2). Therefore, we tried to refine the information of the DCA score provided for OntoGPT by adding more specific definitions (Supplementary Material), which improved the success rate to 61/120 (51%). However, in many cases, the LLM had problems to assign only one parameter, which was why we provided the possibility to assign multiple labels. Lastly, this approach proved successful, OntoGPT was able to assign all genes but 8 out of 130 (95.4%) to at least one parameter. In comparison to the manually curated list, 68% were assigned concurringly, but the justification provided by OntoGPT often was more accurate than the manual assignment.

The gene clusters that were attributable to the respective DCA parameters are shown in Figure 5B. However, for 9 genes, OntoGPT failed to assign a label which are not shown in the figure. As a result of this assignment task, 36 genes were attributed only to D, 24 genes only to C, and seven genes only to A. 20 genes were applicable for both D & C, and 16 genes for D & A. A total of 8 genes matched the description for all parameters, among them TNF, IL1B, CCR7, and CCL20.

## Discussion

The aim of this project was to elucidate the validity of the IBD-DCA score in pediatric IBD with regard to its efficacy in determining the degree of mucosal inflammation by using tissue transcriptomics in an AI-assisted approach. Our results show that the underlying disease entity does not mainly account for the observed transcriptomic differences and that rather inflammation itself is the most prominent discriminator, as the IBD-DCA score does clearly discriminate between high and low inflammatory conditions. We could also demonstrate that all the 130 DEGs are closely connected to each other in a protein-protein interaction network. Moreover, we identified eleven hub genes at integral positions within that network, each playing a pivotal role in immune regulation. By use of the LLM OntoGPT that was recently published,^24^ we were also able to assign each of the 130 DEGs to at least one of the parameters that constitute the IBD-DCA score—a circumstance that supports the accuracy of this score in determining the degree of inflammation in pediatric IBD. However, these results are of an exploratory nature and should not be interpreted as objective validation.

Recently, tissue transcriptomic analyses have already been performed separately for pediatric patients with CD^26^ and UC.^27^ Both datasets revealed distinct transcriptomic signatures that account for the activeness of the disease. In ileal biopsies of therapy-naïve children with CD, a particular involvement of Th17 cells has been demonstrated, which is further positively supported by distinct transcriptomic alterations of epithelial cells and monocytes in the affected tissue samples. Here, our data support the findings of Ashton et al., as we were also able to identify similar genes like *SOCS3*, *NOS2*, *S100A8/9*, or *IL1B*.^26^ Interestingly, the results of the UC study of Pang et al. differ to a much greater extent, which might be explained by the fact that only rectal biopsies from only five patients were used.^27^

In that context, the importance of integrate modern molecular-biological techniques like Next-Generation Sequencing (NGS) into the diagnostic process will represent an important step towards the aim of molecular remission^28^ and is highlighted by the study of Siebert et al. who have demonstrated that transcriptomic signatures of Th17 cells persist in mucosal biopsies of IBD patients with endoscopic healing. In that line, Argmann et al. recently published a newly developed molecular inflammation score (bMIS) using biopsy tissue transcriptomics and validated that score in four independent adult as well as pediatric IBD cohorts.^29^ Notably, the bMIS harbours the same pathways like Oncostatin M, Interleukin-17, TNF-alpha, and Interleukin-1-signalling that were identified by Ashton et al. as well as by our group. In that context, the bMIS score was even able to predict the risk of future inflammatory flares in UC patients with endoscopic and histological remission. Moreover, Argmann and colleagues highlight the observation that the biopsy localization and degree of tissue inflammation have a much higher influence on the transcriptomic signature than the underlying disease entity.^29^ Although we used a slightly different approach by using biopsies from sites with the most prominent inflammation, our results support a similar conclusion. Altogether, these data highlight the potential that transcriptomic profiling of disease activity harbors with regard to patient monitoring and tailoring therapeutic options.

However, there are several limitations that have to be discussed. First, all of the included patients with UC displayed a high degree of inflammation. There were no patients with low DCA scoring results, which limits score validation for the UC cohort. Second, the seven patients from the CD—low DCA—cohort had a more prominent inflammation in localizations elsewhere than the specimen that was previously selected for RNA sequencing. Although this approach gave us the opportunity to study the transcriptomic differences in low versus highly inflamed tissues, this fact also might explain the noticeable discrepance between DCA score and (endoscopic) activity indices as well as calprotectin. Further studies, ideally using a multicentric design, are warranted to definitively validate the IBD-DCA score for pediatric IBD.

Also, as a tertiary pediatric health care center, we were not able to exclusively include therapy-naïve children. However, the majority of patients already receiving an immunomodulatory regimen were assigned to the high DCA group and displayed increased disease activity nevertheless. Moreover, all recruited patients were naïve to biologicals. Methodically, our results represent only a section of the involved genes (modules) and pathways, as we have only used a panel of immunologically relevant genes and an RNA sequencing approach. Therefore, the contribution of other pathways that are involved in fibrosis or autophagy, both also important factors in IBD, could not be examined. Applications like spatial transcriptomics that are able to reveal transcriptomic patterns within their spatial context will overcome that important hindrance.^30^

## Conclusion

In conclusion, our data show distinct transcriptomic signatures of mucosal inflammation, which is irrespective of the IBD entities CD or UC. The IBD-DCA score effectively discriminated those high or low inflammatory profiles and was applicable to both CD and UC alike. Therefore, our data highlight the importance of a validated and uniformly applicable histological score in the process of integrating transcriptome data into the diagnostical process. Although the existing body of evidence in the current literature clearly points in that direction, there is an urgent need for further multicentric prospective studies with well-defined patient cohorts. Moreover, experts in the field will have to define the most significant transcriptomic signatures of mucosal inflammation before these technologies can be used in daily patient care.

## Supplementary Material

otag023_Supplementary_Data

## Data Availability

All data relevant to this study are available in the main document or in the supplementary materials.
