# Supplementary material for: Inflammation significantly alters mucosal transcriptomic signatures in pediatric inflammatory bowel disease
Source: Crohns Colitis 360. 2026 Mar 28;8(2):otag023. doi: 10.1093/crocol/otag023 (PMC13099381; doi:10.1093/crocol/otag023)
Supplement: otag023_Supplementary_Data [file otag023_supplementary_data.zip › Supplementary Table legends.docx]

Supplementary Table legends:

Supplementary Table 1: List of DCA scoring results for each patient and localization.

Supplementary Table 2: List of Differentially Expressed Genes.
