# Supplementary material for: Inflammation significantly alters mucosal transcriptomic signatures in pediatric inflammatory bowel disease
Source: Crohns Colitis 360. 2026 Mar 28;8(2):otag023. doi: 10.1093/crocol/otag023 (PMC13099381; doi:10.1093/crocol/otag023)
Supplement: otag023_Supplementary_Data [file otag023_supplementary_data.zip › Supplementary Material-.docx]

Table of contents

[Supplementary Figure 1 2](#_Toc174105015)

[Supplementary Material 3](#_Toc174105016)

## Supplementary Figure 1


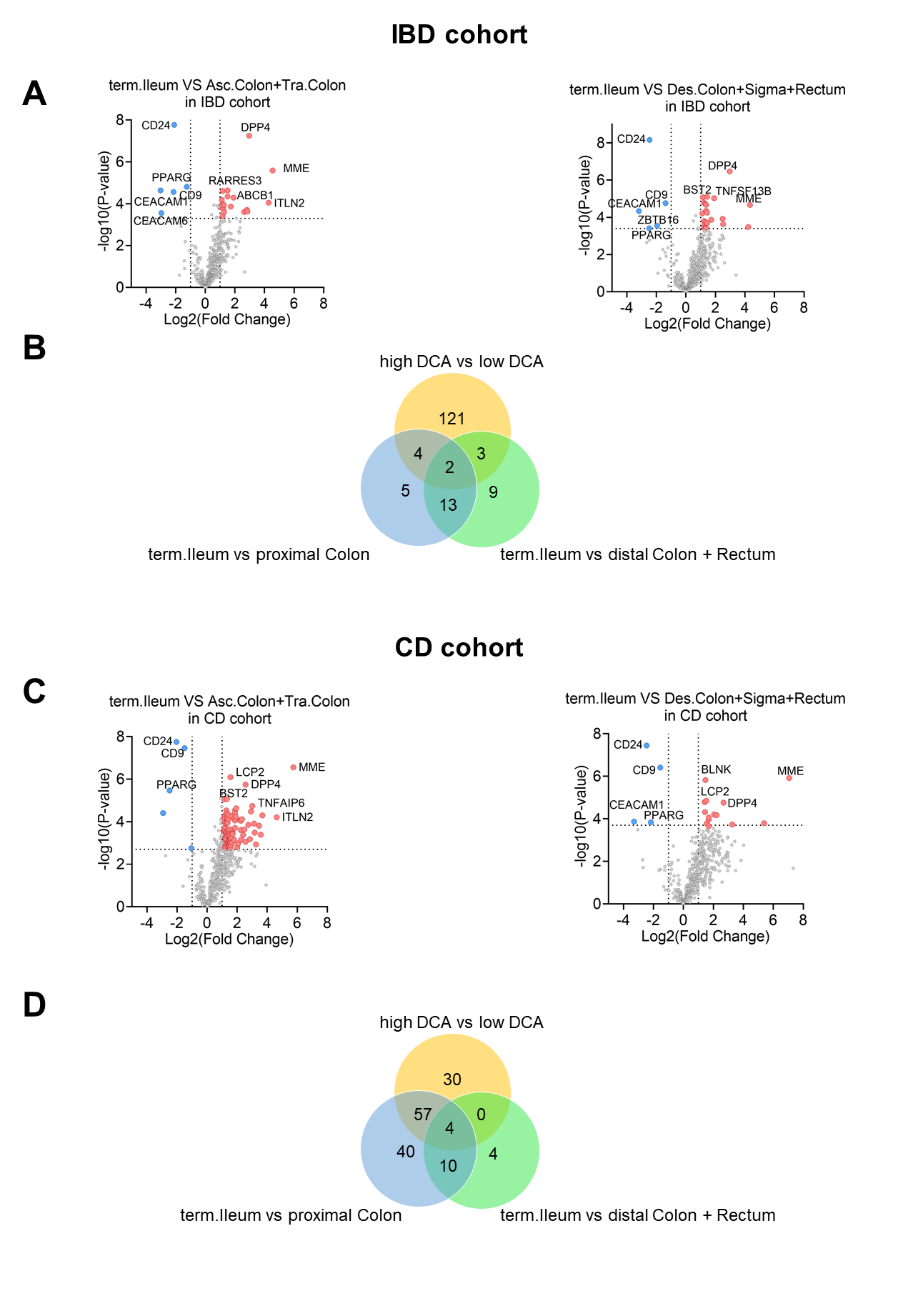


**Supplementary Figure 1. Mucosal RNA expression patterns. (A & C)** Volcano plots depict differentially expressed genes when terminal Ileum and proximal Colon (left panel) or distal Colon (right panel) for the entire IBD cohort (A) or only CD patients (C) are compared. Red dots represent significantly elevated expression. The criteria are set as Log FC > 1, adj. p < 0.05. **(B & C)** Venn diagrams show the amount of overlapping genes for the DCA or localization analysis.

## Supplementary Material

**Definitions for OntoGPT:**

Black: Definitions of the DCA parameters provided by Lang-Schwarz C et al. Virchows Arch. 2021 Mar

Yellow: Additional definitions provided by the authors

Parameter A refers to the presence and degree of tissue involvement by neutrophilic granulocytes. A0 is assessed in the absence of neutrophilic granulocytes. The number of neutrophils that are allowed in a normal lamina propria ranges in the literature between 0 and 1. Neutrophils are not normally present in the surface or crypt epithelium. Therefore, A1 is assessed for an increase of two or more neutrophils in the lamina propria in one high-power field (HPF) or one or more neutrophils in the epithelium (as in cryptitis or neutrophils in the surface epithelium). To reach the intestinal lumen, neutrophils must first exit the blood vessels, migrate across the lamina propria and finally cross the epithelial barrier in that order. Therefore, A2 is assigned in the presence of crypt abscesses, erosion or ulceration as they have breaks of the mucosal barrier in common.

Neutrophils = immune cells that are activated first-line in inflammatory processes

Inclusion criteria for Parameter A:

- Receptors or other molecules that expressed on neutrophils (e.g. chemokine receptors, Toll-like receptors)
- Messenger substances that act on neutrophils / granulocytes (cytokine = activating or inhibitory substances, chemokines = substances that form a chemical gradient and navigate immune cells to the inflammation site = chemotaxis)
- Messenger substances (cytokines, chemokines) that are produced by neutrophils
- Signalling pathways that are relevant for neutrophils
- Components that are relevant for neutrophilic function (phagocytosis, production of reactive O2-species [ROS], antigen-presentation on MHC1, NETosis)

Parameter C encompasses crypt architectural distortion as well as elevated lymphoplasmacytic cell count in the lamina propria (including basal plasmacytosis). C0 refers to mucosa without chronic changes, i.e. absence of elevated lymphoplasmacytic cell count and absence of crypt architectural distortion. C1 refers to crypt distortion and/or mildly elevated lamina propria lymphoplasmacytosis (mildly more lymphocytes and plasma cells than in the normal mucosa). C2 necessitates a marked elevated lymphoplasmacytic cell count in the lamina propria regardless of the additional presence of crypt distortion. A marked basal lymphoplasmacytosis is also assessed as C2.

Lymphoplasmacytosis = Presence of lymphocytes (T- or B cells) or plasma cells (B cells that produce antibodies)

Inclusion criteria for Parameter A:

- Receptors or other molecules that expressed on lymphocytes or plasma cells (e.g. chemokine receptors, Toll-like receptors)
- Messenger substances that act on lymphocytes or plasma cells (cytokine = activating or inhibitory substances, chemokines = substances that form a chemical gradient and navigate immune cells to the inflammation site = chemotaxis)
- Messenger substances (cytokines, chemokines) that are produced by lymphocytes or plasma cells
- Signalling pathways that are relevant for lymphocytes or plasma cells
- Components that are relevant for lymphocytes or plasma cell function [(secretion of antibodies (plasma cells), antigen-presentation on MHC1 (T cells), antigen-presentation on MHC2 (B cells)]

Parameter D is used to estimate the overall extent of mucosal abnormalities, regardless of whether they represent architectural distortion, chronic inflammation or active inflammatory infiltrates.

In general, mechanisms that navigate immune cells to inflammation sites (chemotaxis, migration, integrin receptors, extravasation, adhesion) are related. Also cells, that are normally present in the mucosa (e.g. dendritic cells, tissue macrophages) are related to D.

But, D is the most generalistic marker, every gene that is some what immune-related can be accounted for that parameter, especially if it does not fit A or C. Also, markers that are ubiquitiously expressed in the immune system can be attributed to D.
